# Tempest in a teapot? Toward new collaborations between mainstream policy process studies and interpretive policy studies

**DOI:** 10.1007/s11077-020-09387-y

**Published:** 2020-05-22

**Authors:** Anna P. Durnová, Christopher M. Weible

**Affiliations:** 1 Institut für Höhere Studien - Institute for Advanced Studies, Vienna, Austria; 2 School of Public Affairs, University of Colorado Denver, Denver, USA

**Keywords:** Policy process research, Interpretive policy studies, Public policy, Discourse, Knowledge, Approach to research

## Abstract

“Tempest in a teapot” is an idiom that refers to a problem that has been blown out of proportion, which is how we see the supposedly divisive relationship between two research traditions: mainstream policy process studies and interpretive policy studies. In this commentary, we explore both research traditions, comparing and contrasting their views of public policy and policy processes, uses of theories, and approaches to research. Our aim is not to unite them or reject points of debate. Instead, we offer strategies for more productive collaborations, including side-by-side research, integrative research, engagement in constructive discussions of research techniques, and applied research.

## Introduction

A “tempest in a teapot” is a problem that has been blown out of proportion. This idiom encapsulates ongoing miscommunications and animosities between mainstream and interpretivist policy scholars. The difference between these traditions has generated conflict in policy studies for decades, with some of the divisions passed down through generations. Of course, conflict can fuel learning and, if properly handled, resulting in more robust and productive relations. Yet, the opposite seems to have happened. Our purpose here is not to relive and reiterate these old debates—after all, let bygones be bygones. Instead, we compare and contrast both research traditions. While we see differences in their orientations and methodologies, we also see goal similarities given their distinct foci and emphases. Moreover, collaboration offers the potential to conduct research together in order to advance knowledge and contribute to society.

This commentary attempts to evenhandedly describe these two traditions for both new and experienced policy scholars.^1^As two scholars in each of these two traditions, we wrote this commentary jointly in order to develop a common conceptual terminology and an understanding of research approaches. Two people cannot summarize every aspect of both traditions, but we trust that our interpretations are not too narrow and not too far off. While we maintain our lofty aspirations in offering a better understanding of both traditions and ideas for collaborations, our more humble ambition is to provide a shared language and a better understanding of more fruitful communications.

### Mainstream public policy studies and interpretive policy studies

Let us start with how both traditions might study the game-of-chess as if it were a policy-related phenomenon. The mainstream policy scholar would approach the game from two perspectives. From the first perspective, and among those who practice “mainstream policy analysis,” the pros and cons might be analyzed in order to determine the next move for one of the chess players. They then might forecast the risks and benefits of different moves and communicate these tradeoffs back to the chess player to support decision-making. Alternatively, these mainstream policy analysts might evaluate a prior move. Was it the right move? What were the benefits and costs of that move? From the second perspective and among those who practice “mainstream policy process studies,” understanding how all the chess pieces move and interact over time might be important. The game becomes complex because of the rules and the varied strategies that chess players use, prompting the need for theories to capture the dynamics. The resulting insights would then be communicated back to the chess players as general understandings about the nature of the game and ways of playing.

The interpretive policy scholar might start from a point of curiosity about the rules of the game and the ways these rules are contingent as constituting part of a particular culture, social group, or geopolitical context. They might then understand how the contingency of these rules oppress and limit options to make moves and develop strategies. Then they might uncover the reasons some potential players are discouraged from playing. They might explore the pieces as objects and their movements as acts—both of which would be embedded with the meaning of values, beliefs, and feelings. Knowing that meaning is conveyed through situated interactions, they might analyze how the players interrelate through language. Additionally, recognizing the subjective orientations of the players and themselves, interpretive policy scholars might immerse themselves in the game and interact with the players to develop an understanding of how this one game-of-chess is being played. Through this one game, they might try to understand how the rules were and are established via situated interactions of players. Part of the interpretive policy scholar’s agenda involves questioning the establishment of these rules and the connections between players and non-players.

This simple game-of-chess analogy succinctly portrays mainstream policy analysis, mainstream policy process studies, and interpretive policy studies. It shows that these traditions can offer complementary ways of understanding the same phenomenon. We provide brief descriptions of these traditions below.

#### Mainstream public policy studies

We use the term “mainstream public policy studies^2^” to encompass both mainstream policy analysis and mainstream policy process studies, excluding interpretive policy studies. We consider “mainstream” an apt description because it has been dominant in shaping many of the norms that ostensibly run counter to interpretive policy studies. We use the term “mainstream policy analysis” to refer to the area of study that offers practical or client-oriented advice in evaluating past decisions or assessing future decisions (Bardach and Patashnik 2019; Weimer and Vining 2017). Mainstream policy analysis is often associated with decision-making tools (benefit–cost analysis, distributional analysis, etc.). “Mainstream policy process studies” represent the area of study that describes and explains the varied interactions that embed and surround public policy (Weible 2018; Cairney 2011). While mainstream policy process studies can pivot around a single public policy, encompass multiple policies across space or time, deal with the politics and impacts surrounding public policies, or focus on substantive policy issue (and many public policies therein), they involve a range of factors that include, but should not be limited to, actors and organizations, political behaviors, events, contexts/settings, and outcomes. Whereas mainstream policy studies are associated with tools that help inform policy decisions, mainstream policy process studies are associated with theories that help describe and explain public policy (Lubell 2013; Weible and Sabatier 2018).

#### Interpretive policy studies

We use the term “interpretive policy studies^3^” to encompass the various approaches to investigating public policy through its discursive nature. This means that meaning can be uncovered and can differ in acts, actors, and objects around public policy and in events that happen to public policy. The language used to describe public policies and to discuss or negotiate them shapes who becomes legitimate or powerful and, more generally, how the policy process develops over time. This distinctive way to understand and highlight public policy relates to the capacity of language to deliver contextual information about a situation and to change the situation. Placing that interest in the language above all other inquiry, interpretive policy studies perceive itself in opposition to “positivist policy analysis,” which is considered a form of knowledge oppression because it limits analysis to certain questions and organizational or institutional spaces and prevents the analyst from uncovering the conditions that have established these limits. Interpretive policy studies build on the possibility of multiple meanings and then analyze how these meanings coproduce policy processes, that is, which meanings are attributed by whom and where, thereby seeking to explain what practices and what power structures these specific meanings reveal (Bacchi 2005; Durnová et al. 2016).

We next explore the differences and similarities of these two traditions. We deliberately narrow comparisons of interpretive policy studies to mainstream policy process studies and not to mainstream policy analysis. We do so because mainstream policy analysis is notably different from policy process studies and including both would complicate our commentary. Similarly, we do not discuss the differences between the sub-traditions found within interpretive policy studies but focus on the tradition’s overarching principles and practices.

### Scopes and views of public policy and policy processes

Table 1 compares and contrasts the scopes and views of public policy and policy processes from interpretive and mainstream traditions. Mainstream policy process studies typically view public policies as products and sources of politics or constituting the institutional landscape that shapes and is shaped by political behaviors. Public policies can be viewed as translations of understandings, interests, values, or beliefs (Sabatier 1988). Mainstream policy process studies make a distinction between public policies as either “rules-in-form” or “rules-in-use” that, respectively, represent policies written and adopted by a decision-making venue (e.g., in a statute or regulation) or the regularized behavior of government officials, street-level bureaucrats, or other actors engaged in the practices of government (Ostrom 2005). Mainstream policy process studies then focus on the contexts, events, actors, and outcomes that surround and embed public policies.

**Table 1:** Comparing and contrasting views of public policy and policy processes

	Interpretive policy studies	Mainstream policy process studies
Scopes	Focus on understanding meanings in actors, events, and objects of public policy phenomena. Situate strategies and behaviors in the context of power relations in the sociopolitical context	Focus on describing and explaining public policy phenomena, including (but not limited to) research on stages of the policy cycle and analyzing political behaviors
Views of public policy	View public policies as both rules-in-form and rules-in-use and as manifestations of meanings, which are associated with values, beliefs, emotions, feelings, and power structures	View public policy as both rules-in-form and rules-in-use and as sources and products of politics and translations of understandings, interests, values, and beliefs
Views of policy processes	View policy process as involving the study of meanings in, and of, the interactions surrounding public policies, in particular actors, objects, and language, wherein there is a heavy emphasis on discourse and underlying power structures	View policy process as involving the study of all the interactions surrounding public policies, including actors, events, contexts, and outcomes

For interpretive policy scholars, since public policies are manifestations of meanings that actors create and that can be conveyed through the artifacts of language (Yanow 2003; Bacchi 2005; Torfing 2005; Hay 2011), discourse becomes an important concept and a way of understanding and representing the policy process (Bacchi 2009; Fischer and Gottweis 2012; Dodge and Metze 2017). While actors see and transform the world through discourse, these actors are shaped through the same discourse and can be transformed by it. Interpretive policy studies view the formation of the policy process through semantic categories used in everyday interactions, observed through the use of specific words, arrangement of these words in sentences, narratives, metaphors, arguments and rhetorical figures that frame actors attempting to influence public policies and the intended receivers. The aim of interpretive policy studies is to focus on the conceptual understandings of why public policies emerge in these specific forms.

These traditions share some commonalities. Both traditions focus on public policies to understand governments (although interpretive policy studies will often seek this understanding outside of the usual institutional structures of governments for reasons explained below). Both traditions view public policies as something written or in use that shape outcomes. Both focus on the politics surrounding public policies that involve interest groups, powerful entities, and others. Both aspire to understand the processes through which these policies emerge and expire. However, interpretive policy studies emphasize the power of language more as something that shapes policies while also being shaped by them. Interpretive policy studies want to understand how knowledge is both constructed and performed, who gets a say in the policy process, who is considered a legitimate actor, and who becomes marginalized, silenced, or omitted. Mainstream policy process studies also study power and language but emphasize it less and research it differently. For example, mainstream policy process studies might analyze shifts in the news media discourse as an expression of power and in relation to changes in public policies. Conversely, interpretive policy studies focus more on how language constructs the relation between expressions of power and changes in public policy. As mainstream policy process studies focus less on these underlying structures of language and power, interpretive policy studies have perceived mainstream policy process studies as contributing to concealing power relations and oppressing certain forms of knowledge and, thus, becoming part of the discursive landscape that needs to be analyzed.

### Uses of theories

Table 2 compares and contrasts the uses of theories in the two traditions. Mainstream policy process studies generically use theories as a way to organize inquiry, establish the scope of such inquiry (e.g., types of questions asked), specify assumptions, define concepts, and relate those concepts (e.g., in the form of principles, hypotheses, or propositions). Theories act as platforms for organizing research programs that enable collaboration among groups of scholars. This supports the production of simultaneous theory-guided research applications in different locales, in different points in time, on different topics, through different methods, and by different researchers that can inform and contribute to knowledge, which can then be used to revise and update theories. Theories, thus, become continuously revisited and updated reservoirs of knowledge about policy processes. Theories also help mitigate subjectivity and bias of the researcher (see the next section).

**Table 2:** Comparing and contrasting use of theories

	Interpretive policy studies	Mainstream policy process studies
Uses of theories	Provide insight into value orientations and value constitutions and use reflexivity in the systematic back and forth between field and theories	Provide a platform (shared vocabularies and assumptions) for establishing research programs, serve as reservoirs of knowledge, simplify and guide research, and organize inquiry around relational forms (e.g., hypotheses)
Relational forms	Include hunches or propositions, which highlights the interdependence of what is studied and who studies it. Include also grounded theory, which emphasizes porosity between field and understandings. Use reflexivity as a way to assess personal or topical biases that necessarily emerge in any analysis of public policy phenomena	Include hypotheses, propositions, and principles for varied purposes, such as to refute or confirm expectations and to posit explicit causal or non-causal associations in helping to organize inquiry. Adopts a major goal of generalizability of theoretical arguments given contextual conditions
Major theoretical approaches	Include (but is not limited to) argumentative policy analysis, interpretive policy analysis, deliberative policy analysis, poststructuralist policy analysis, critical policy studies, narrative policy analysis, and rhetorical policy analysis	Include (but is not limited to) punctuated equilibrium theory, multiple streams framework, institutional analysis and development framework, the advocacy coalition framework, diffusion of innovation, policy feedback and historical institutionalism, social construction and policy design, narrative policy framework, and ecology of games framework

The relational forms (such as relating concepts in hypotheses or propositions) posited in theories vary in their utilization within mainstream policy process studies.^4^ For some, these relational forms state associations to confirm or refute. For others, relational forms serve direct inquiry and organize and communicate the presentation of findings. Sometimes relational forms specify causal drivers and emphasize processes (mechanisms) or variances (effects). When a causal argument is made, a theory usually offers the rationales underlying the relationship, often tied to a model of the individual (e.g., what is assumed about an individual’s motivations and cognitive abilities). Other relational forms are more prescriptive in specifying the conditions associated with the likelihood of a phenomenon to exist or are descriptive by positing patterns. Sometimes these relational forms direct researchers to specify the context on which they depend. In this way, relational forms are stated with broadly defined concepts that are adaptable to different contexts given the rationale or logic established in the theory.

In interpretive policy studies, there is a deliberate absence of hypotheses. Concepts and their interrelations, as we usually find them in mainstream policy process studies, also exist in interpretive policy studies and can be understood both as associations to confirm or to refute and as guideposts to organize the analysis. What differs in interpretive policy studies is that they are created from the inquiry and analysis in the field rather than previously derived from a theory. The relation between the use of theories and the way to proceed in the inquiry in interpretive policy studies can be summarized under the “logics of inquiry,” a term that encompasses norms and strategies for guiding interpretive scholarship (Schwartz-Shea and Yanow 2013). Two key terms that summarize aptly the logics of inquiry are “intersubjectivity” and “interdependence.”

“Intersubjectivity” means that knowledge emerges from the interpretation of interactions between acting subjects, objects, or texts and, as such, it can be accessed only contextually (Durnová 2015). It is also not something that exists independent of the researcher or as something to be found.^5^ These interactions are studied through all kinds of practices. As a consequence, interpretive policy studies scholars often refer to such contextualization as situated interactions. An important aspect of situated interactions is the view of interpretive policy scholars that they, as researchers, are a part of such interactions and that their observational position (e.g., social, cultural, and national origin) is part of the analysis of their research. Thus, interpretive policy scholars practice a degree of self-awareness in collecting and analyzing data in what is called “reflexivity.”

Interdependence relates to the way theories in the interpretive tradition layout assumptions about the policy process (Hajer and Wagenaar 2003). Theories informing interpretive policy studies presuppose contingent formations of social phenomena and layout possibilities of studying and following that contingency. They transcend with what they see as the objectivistic, reductionist, and rationalistic bias of modern social science theories that shape understandings of the surrounding world (Torfing 2005) and highlight the (socially) constructed character of norms, values, symbols, identities, and knowledge paradigms. While theories in interpretive policy studies have explanatory value, their aim is not to establish covering laws or to reveal the intrinsic causal properties of social objects. Theories in interpretive policy studies aim, instead, to understand why particular policies were constructed, stabilized, or transformed (Torfing 2005) and how this happened.

### Approaches to research

The major differences between mainstream policy process studies and interpretive policy studies can be found in how they conduct research (see Table 3). They differ in their ontological and epistemological orientations, in assessing quality, in handling human bias, and in treating generalizability. Yet, there are also similarities. For example, both care about human bias but handle it differently, and both care about quality science but gauge it differently.

**Table 3:** Comparing and contrasting approaches to research

	Interpretive policy studies	Mainstream policy process studies
Ontological & epistemological orientations	Assumes a constructive ontology and interpretive epistemology and followed by consistent methodologies and method	Focuses heavily on methodology and methods and rarely emphasizes ontological or epistemological orientations
Dealing with human bias of the researcher	Accepts reflexivity in assessment of data collection and data analysis and embraces human bias during research and in discussing the results	Mitigates human bias through specifying all assumptions and steps in the research process, including clear conceptual definitions and measurement usually embedded and guided by theory
View of generalizability	Emphasizes particularities or singularities of a case, however, might address conceptual inference (relationships between categories)	Emphasizes generalizability with the goal of teasing apart localized insights from those that generalize over time or space
Gauging quality	Judges research by its credibility, dependability, and confirmability and overall transparency and clarity	Judges research by its reliability and validity and overall transparency and clarity

#### Philosophical foundations

Interpretive scholars moor their philosophy of science to a constructive ontology and interpretive epistemology (Dodge 2015; Schwartz-Shea and Yanow 2013). Constructive ontology means that public policy phenomena are constructed through meanings assigned to them by various actors, and interpretation is then seen as the suitable (epistemological) means to reveal the rules and operations of that construction. Under this philosophical orientation, the construction will always interrelate dynamically with structural conditions and agencies challenging them. Interpretive policy studies are oriented as consciously anti-positivist and conceive “positivism” as a form of procedural oppression that obscures hierarchies between included and excluded actors and the corresponding creation of meaning and established understandings. Mainstream policy process scholars are then assumed to be part of the group representing “positivism” and “the other.”

In turning mainstream policy process scholars into the other, depictions of their philosophical orientations have become exaggerated and erroneous caricatures. These depictions of mainstream policy process scholars’ philosophy of science have included the following: researchers are without presuppositions or biases and perceive the world as independent of them; causality is akin to “hitting a cue ball on a pool table”; discovering covering laws and causal explanations that span all contexts is the sole purpose of research; context is irrelevant; the world is stable; public policies signify objective instruments of rationality rather than translations of beliefs and values or products of politics; and conceptual measures are objective representations of the truth.

While these caricatures are unsubstantiated or exaggerated, a question then arises: what are the philosophical foundations of mainstream policy process studies? To such a question, the strawman caricature described in interpretive policy studies needs updating and corrections, but it is beyond the scope of this paper to detail what mainstream policy process scholars believe and practice as their philosophy of science. We speculate (without much of a basis beyond our own observations) that most mainstream policy process scholars recognize their presuppositions and biases, the lack of objectivity in their measures, the inherent challenges in any attempt to specify causality and, hence, the emphasis on associations and patterns and, at best, probabilistic relationships, the importance of contexts, the value of quantitative and qualitative approaches, dynamism of policy processes, and public policies are translations of beliefs and values and, hence, reflect and influence power and politics.

#### Dealing with the researcher’s human bias

The two traditions differ in dealing with human biases, which we describe through another analogy. Imagine how scholars from both traditions would grocery shop. Mainstream policy process scholars, concerned about their own cognitive limitations and biases, might use a shopping list. Analogous to the use of theory, a shopping list might help guide what to pay attention to and what to ignore and, thus, guard against researchers’ presuppositions to shop only in one aisle or on a whim of hunger. Behind this shopping list might be one or more chosen recipes. The extent that researchers would shop beyond their list is contextually dependent—sometimes they will shop beyond the list and other times not. Mainstream policy process scholars would update and modify their grocery lists or have multiple lists depending on their values, objectives, and the store visited. Interpretive policy scholars are also concerned about the subjective nature of their research but embrace it. Interpretive policy scholars might shop without a grocery list. They might view a grocery list as both oppressive on their shopping choices and as a false way to mitigate their biases. Their purpose in shopping would be to cook a meal that portrays their interaction with, and the identity of, the grocery store. When revisiting a grocery store, they might draw insights from prior shopping experiences not as a list but as conceptual suggestions about how next to grocery shop.

In dealing with human bias, mainstream policy process scholars want their publications to hold as much detail as possible about the conduct of the research because this is a reflection of clarity and transparency and a means of mitigating human bias. For example, they might want to see the grocery list in the analogy above (i.e., the interview questions or survey questions used in research). This then becomes a common criticism from mainstream policy process studies of interpretive policy scholars, that is, interpretive scholars are not clear enough in their data collection and analysis. On the other hand, interpretive scholars want to see in their publications not a shopping list but explicit acknowledgment of the dynamics between the researcher and the phenomenon studied in a reflexivity assessment. Interpretive scholars do not treat subjective nature and human bias as limitations but rather as a legitimate part of research and a reason to conduct research. Assessing reflexivity means expressing awareness of the situated relationship between the studied phenomenon and the researcher. This assessment might include the structural conditions that affect the inquiry, especially the know-ability of the phenomenon (Shehata 2006). A substantial part of this reflection includes expressions of the contradictions and limitations affecting the choice of the researcher and the methods used. Expressing reflexivity in the research, thus, becomes a statement of clarity and transparency. To return to our grocery store analogy, interpretive policy scholars want their publications to show how shopping and buying food has contributed knowledge about the grocery store, how hunger and nutritional needs of the researcher interacted with the shopping experience, and how—knowing all that—we can interpret the food in the store and how that food might relate to particular practices of shopping and cooking. This assessment usually appears not in the methods section but is expressed throughout the publication.

According to interpretivists, the errors of using theories without taking into account their ontological background (i.e., analogous to the shopping list above) show a lack of reflection on the discourse that springs from the tradition of positivist objectivism. Reality, as depicted in a positivists’ way of thinking, is simply “out there” and can be known through purely objective rational procedures. That is why they perceive mainstream policy process scholars as using the same theories repeatedly without considering contexts. Such perceptions conflict with interpretivists, who embed knowledge as a part of all sorts of social relations and interactions with the context and with power dynamics that affect the production of knowledge. Yanow (2003) considers both contextual information from interviews and the field that is often used to inform more of the “science” phase of mainstream policy process studies as problematic. For interpretivists, engaging with the field is already part of the scientific process.

This importance of a philosophical rigor within the use of theories helps explain why interpretivists do not visualize theories as tools in a toolbox. They want to define their tools after they have seen the policy problem they are analyzing. Toolbox imagery, from an interpretive perspective, can be limiting because it might divert researchers from the start toward categories that misrepresent the experience from the field. This further explains the related perception among interpretivists that the use of theories as practiced by mainstream policy process scholars limits their understanding of the phenomena studied. Their use of theories also may obscure alternative narrations of the policy problem and meanings held by marginal social groups not considered in the theories.

However, mainstream policy process scholars neither assume that reality simply exists “out there” independent of them nor argue that their research is objective. Indeed, the explicit statement of research methods in publications signal mainstream policy process scholars’ uneasiness with the lack of objectivity in their procedures and the potential biases their presuppositions might bring to their research. Moreover, theories (i.e., the grocery lists) in mainstream policy process studies are not static or applied blindly. Theories can incorporate decades of research and experience and, thus, are updated and adapted to new contextual situation. Indeed, applying a theory thoughtlessly without contextual considerations is bad science. For example, a theory might offer a broadly defined concept that enables the researcher to adapt and apply it appropriately to a given setting. To make this happen, some mainstream policy process scholars might apply various forms of applied scholarship to design, pretest, or ground-truth their data collection instruments (Van de Ven 2007), which is a part of the scientific process. Mainstream policy process scholars also use the framework-theory distinction to conduct their research comparatively and to incorporate context (Ostrom 2005). Through this distinction, a framework provides a portable and very general platform in the types of questions asked, concepts and shared language, and general relations among them. A theory then might incorporate a subset of the framework’s concepts, and maybe additional concepts relevant to a case, to help understand and explain a particular situation. In this regard, frameworks provide portability across contexts and theories provide the adaptability to a particular context. Mainstream policy process scholars might not immerse themselves in the field as much as interpretive scholars, but they certainly incorporate it into their research.

For mainstream policy process scholars, the use of theories (generally stated) helps bolster or refute parts or all of their knowledge through seeking errors and making corrections, finding surprises or confirmations, and learning from past experiences. By employing more than one theory, mainstream policy process scholars recognize and mitigate the oppressive way of thinking imposed by any one theory or approach. Thus, they use theories as lenses to guard against seeing the world from a biased or singular perspective. Of course, they maintain their common sense and instincts, but they also approach their phenomenon from distinct vantage points as suggested from different theoretical perspectives. Hence, for mainstream policy process scholars, theories (akin to tools in a toolbox) provide a means for critical thinking, a freedom to approach research using multiple perspectives, and a platform to build knowledge and learn from mistakes.

#### Generalizability

The two traditions differ in how they approach generalizability. A common perception among mainstream policy process scholars is that interpretive policy studies are plagued by relativism. Indeed, interpretive policy scholars view the conduct of comparative research and the practice of finding generalizable lessons as antithetical to their goals. Constructive ontology of interpretive policy studies underscores the situated character of actions and contingency, which downplays possibilities of generalizations. Interpretive policy scholars endeavor, instead, not to generalize but to show how actions, actors, and objects are situated with meaning, by whom they are situated, and how this might affect the way the meanings are understood. Interpretive policy scholars approach the issue of generalizability by exploring how insights are constructed, reflect power structure, and omit certain knowledge. Interpretive policy scholars might even ask why society highlights generalizations as the goal of scientific expertise. From a different perspective, defining generalizations as repeated patterns of actions or a configuration of actors, interpretive policy studies might offer analyses of repeated contingencies or relations between actors and contexts in different policy areas and in different times or different countries. Interpretive policy scholars might also focus on conceptual inference by drawing conclusions from their data on the relationships between categories (in the meanings of objects, actors, or words) as instances of broader recognizable patterns or features (Williams 2000; Schwartz-Shea and Yanow 2013). For mainstream policy process scholars, generalization (i.e., external validity) is a central component of their research enterprise. This is not about finding “covering laws” that fit all contexts. The challenge for mainstream policy process scholars is separating the particularities that fit to a localized context versus those that generalize across contexts and, when such generalizations occur, to what extent and under what conditions.

While both traditions design their inquiry with the help, and on the basis, of theoretical frameworks, they do it differently. Interpretive ontology assumes a strong connection to the field, assumes that people are meaning-making creatures, and that research subjects and researchers comprise the studied world. Interpretivist might, thus, use “hunches” and questions prior to their fieldwork, but they do not posit hypotheses from them. Hunches are grounded in the research literature and often stem from prior knowledge of related study settings. Most importantly, hunches serve as starting points of inquiry that are designed to be revised after the initial field experiences. Adapting the research goals and approach while it progresses is not just allowed but expected.

#### Gauging quality

Interpretive policy studies and mainstream policy process studies gauge the quality of their research differently. Borrowing from Dodge et al. (2005, p. 295), interpretive policy scholars assess their research based on its credibility (i.e., is the research plausible and supported through the data) and dependability and confirmability (i.e., is the research “fair, unbiased, or coherent by people who are external to the process”). In contrast, mainstream policy process scholars primarily assess the quality of their research based on measures of validity (i.e., accuracy in measurement or removing alternative explanations in research design) and reliability (i.e., related to the consistency in measurement).

## Opportunities for collaboration

The fundamental tenet in the interpretive tradition is to avoid combining “positivist” methodology and methods with “interpretive” ontological and epistemological orientations. However, societal challenges have never been steeper as we increasingly face culture wars and backlashes, threats to democracy, and social change due to a pandemic of historic proportions (Fishkin and Mansbridge 2017; Norris and Inglehart 2019; Offe 2017; Weible et al. 2020). In the face of such global calamity, a shared focus on governments, politics, policies, and related outcomes should be emphasized more than the differences in how both sciences conduct themselves. Indeed, the boundaries that offered the intellectual separation between these traditions, especially in the interpretivist tradition, now need to be jettisoned. Given this situation, we categorize opportunities for collaboration in four ways.

1. *Side-by-side research* Scholars from both traditions could analyze the same topic using their respective methodologies and methods, and insights could then be combined into a written product. For example, a publication on the role of conflict in public policy could offer two distinct analyses, one using interpretive methods and one using mainstream methods. The results from both would then be combined to help inform the conclusions. This approach need not require researchers from both traditions to compromise how they conduct their research; it only requires collaboration in writing up the results in a publishable form.
2. *Integrative research* The two traditions can integrate their research on the same project in sequence or parallel. For example, the Comparative Agendas Project has generated large datasets of agenda items and various types of public policies across countries over time.^6^ Interpretive policy scholars could explore this population of cases as a starting point to begin their research and then provide in-depth analysis of one of the cases. This would provide the interpretive scholar a way to articulate how their case might relate to other cases. Likewise, mainstream policy process scholars could conduct their research based on the findings from interpretive policy studies. In this scenario, an interpretive scholar might discover a number of commonly used discourses in a particular case and setting. Mainstream policy process scholars could then use these discourses as points of departure in conducting large-n quantitative analysis of their propensity across space and time. Obviously, we recognize that these integrative approaches might run counter to some of the goals and norms of both traditions. We are not asking either tradition to compromise their research integrity but rather to accept the integrity of the other tradition. In both examples, science is being conducted and one tradition is neither inferior nor superior to the other.
3. *Constructive comparisons of research techniques* The two traditions share similar foci on policy processes but differ in how they conduct research, ask questions, use theory, and gauge quality. These differences have been viewed as points of separation but can also be viewed as opportunities for cross-fertilization and learning. For example, assessment of quality in the interpretive tradition (credibility, dependability, and confirmability) and reflexivity could be used to improve aspects of mainstream policy process studies. Additionally, mainstream policy process studies might draw inspiration from how interpretive policy studies anchor their research to ontological and epistemological foundations. At the same time, interpretive policy studies can draw insights from mainstream policy process studies in communicating the transparency in their methodology and methods.
4. *Applied research* Given that both traditions seek to inform people outside of academia and to contribute to the betterment of society, both traditions could begin by recognizing that nontrivial problems exist and that academics have an opportunity and an obligation to help inform societal responses. Both traditions should, thus, set aside in consequential concerns about their differences. Leaders and non-leaders, the powerful and the powerless, and the decision-makers and the impacted care little about epistemological or ontological orientations, the value of hypotheses versus hunches, the importance or use of theory, and the criteria for gauging research quality. What they want is useful information that can help them make sense of their past, present, and future. For all scholars of public policy, there is a need to put differences aside in jointly conducting applied research in contributing to society (see Weible et al. 2020).

Several research areas are ripe for implementing these opportunities for collaboration. This includes analyzing discourse and stories using interpretive approaches with the Narrative Policy Framework (Jones and Radaelli 2015; Dodge 2015) or with the Social Construction Framework (Barbehön 2020). Similar effort could explore Discourse Coalitions (Hajer 2005) and the Advocacy Coalition Framework (Jenkins-Smith et al. 2017) or the use of language in shaping rules and behavior (Hay 2011; Ostrom 2005). More research could also be conducted on implementation (Maynard-Moody and Musheno 2000). Additionally, both traditions study political conflict that could be integrated (Weible and Heikkila 2017; Dodge and Metze 2017). Finally, one omission in the study of human behavior in both traditions has been the role of emotions, herein, interpretive scholars have begun to develop insights (Durnová and Hejzlarová 2018; Durnová 2018), and this effort could be supplemented with mainstream methodological techniques.

## Conclusion

This commentary seeks to reorient discussions about both mainstream and interpretive policy traditions toward more constructive dialogues and collaborations. Although differences exist and persist, these two traditions are not as polarized as often presented, and they can offer tangible benefits to each other.

Both traditions aspire to understand policy-related phenomena, but they differ in their approaches. Mainstream policy process studies focus more on questions of generalizability and often use theories to build and advance knowledge. Interpretive policy studies focus more on underlying or emerging power structures that shape discourse that then reveals those power structures. Even though mainstream policy process studies contextualize their research, interpretive policy scholars immerse themselves more in the field and adapt their research accordingly. While mainstream policy process scholars might mitigate effects of presuppositions through theories and transparency in their methods, interpretive policy scholars might practice reflexivity while embracing their relationship with their research subjects. Both traditions care about the quality of their research but gauge it differently.

There are untapped opportunities for these traditions to collaborate in conducting side-by-side research, integrating research, constructively comparing research techniques, and applying their scholarship jointly to better society. Collaborations between these traditions could be fostered if mainstream policy scholars would accept a broader definition of social science and if interpretive policy scholars would avoid forcing divisions based on ontological and epistemological orientations. Possibly the best way to help these traditions work together is for scholars to focus on their mutual understandings of methodology and methods in approaching public policy in order to conduct both sciences more transparently and to strengthen their societal pertinence.

## Footnotes

1 There have been attempts to compare interpretive policy studies and mainstream public policy studies (deLeon 1998; Fischer 1998; Lejano and Leong 2012; Weber 2004) and integrate them (Lin 1998; Jones and Radaelli 2015; Boswell and Corbett 2015). However, none of these past efforts have compared and contrasted the terminology and research approaches in both traditions, especially with a focus on mainstream policy process studies and interpretive policy studies. Additionally, one conclusion from past debates has been that, while the two traditions should communicate more, combining them is impossible (Dodge 2015, p. 366), a position that this commentary refutes. Further, one argument claims that interpretive approaches might be more pronounced in Europe than in North America, at least in the study of public policy, but we do not address the validity of such an argument in this commentary.

2 Scholars in the field may use different terms to denote the field as a whole and its subfields. In this essay, we use “mainstream public policy studies,” which is often called “policy studies” or “policy sciences,” excluding interpretive policy studies. Mainstream public policy studies are broad and deep in scope. Since our commentary cannot cover every angle, we’ve chosen to focus on a major portion of mainstream policy process studies. For depictions that overlap with the one in this commentary yet differ in their portrayal, see Clark (2002), Cairney (2011), Knill and Tosun (2012), and Howlett et al. (2009).

3 Interpretive policy studies build on a number of approaches or sub-traditions. Reviewing this literature is beyond the scope of this commentary (see the overview in: Fischer et al. 2015). The diversity within interpretive policy studies is evident by the different labels (such as “argumentative policy analysis,” “discursive approaches,” “deliberative policy analysis,” “poststructuralist policy analysis,” “interpretive policy analysis,” or “critical policy studies”). We use the term “interpretive policy studies” to encompass all these approaches because it is also a term used in both in European and North American scholarship to describe analyses of language and discourse in public policy.

4 We use relational forms given the diversity in how scholars in mainstream policy process studies posit relations among concepts. Sometimes they use hypotheses, principles, and propositions and so forth (Weible 2018).

5 This definition of “intersubjectivity” in interpretive policy studies differs from that in mainstream policy process studies, wherein the term is associated with tests of reliability and consistency in interpreting (e.g., coding or analyzing) the same phenomenon by two or more individuals (Straits and Singleton 2018).

6 See www.compartaiveagendas.net.
